# Translation, cross-cultural adaptation and validation of the Portuguese version of the DYMUS questionnaire for the assessment of dysphagia in multiple sclerosis

**DOI:** 10.1186/2193-1801-2-332

**Published:** 2013-07-22

**Authors:** Déborah S Sales, Regina MP Alvarenga, Claudia CF Vasconcelos, Roberta G Silva, Luiz CS Thuler

**Affiliations:** Federal University of the State of Rio de Janeiro (UNIRIO), Rio de Janeiro, RJ Brazil; Department of Speech and language Pathology, Paulista Estadual University, Marília Campus, São Paulo, Brazil; Coordination of Clinical Research - National Cancer Institute - INCA, Rio de Janeiro, RJ Brazil; Pós-Graduate Program in Neurology, University Hospital Gafree Guinle, Street Mariz e Barros 775 – 2 floor, Tijuca, Rio de Janeiro, RJ Brazil

**Keywords:** Multiple sclerosis, Dysphagia, Swallowing dosorders, Questionnaire, Validation, Reliability

## Abstract

Translation, cross-cultural adaptation and validation of the DYMUS questionnaire for the assessment of dysphagia in multiple sclerosis. The original English version of the DYMUS was translated using the forward-backward technique, cross-culturally adaptated, pilot-tested in 40 patients, and then applied to 100 multiple sclerosis patients to assess the reliability and construct validity. Construct validity was assessed by Mann–Whitney test and Spearman’s correlation coefficient (rs). The internal consistency of the questionnaire was evaluated using Cronbach’s alpha coefficient and inter-item correlation. DYMUS-BR internal consistency was high (Cronbach’s alpha= 0.72); Cronbach’s alpha was 0.65 for the ‘dysphagia for solids’ subscale and 0.67 for the ‘dysphagia for liquids’ subscale and positive inter-item correlations was found between all items, except for weight loss question. Significant association (p<0.001) and correlation (rs = 0,357; p = 0.01) was found between DYMUS-BR and dysphagia self-assessment. The DYMUS-BR questionnaire maintained the characteristics of that originally described, demonstrating to be a reliable, valid, easy and consistent tool to be used by health professionals for preliminary selection of Brazilian MS patients who need more specific instrumental analyses of swallowing.

**Electronic supplementary material:**

The online version of this article (doi:10.1186/2193-1801-2-332) contains supplementary material, which is available to authorized users.

## Background

Oropharyngeal dysphagia in Multiple Sclerosis (MS) is a relatively common symptom, occurring in 33 - 43% of MS patients (Hartelius & Svensson 1994; Prosiegel et al. 2004; Terré-Bolliart et al. 2004). It can increase significantly with disease progression; impairment in the cerebellar (Poorjavad et al. 2010; De Pauw et al. 2002) and brainstem function systems (De Pauw et al. 2002; Leder et al. 1998). The impact of dysphagia is significant, reducing the quality of life and increasing risk of dehydration and aspiration pneumonia in this population. These complications can be prevented through a rapid screening protocol for the risk of dysphagia in these patients (Poorjavad et al. 2010). In this scenario, the identification of dysphagia symptoms in early stages of MS allows referral to more specific investigations by specialized professional, which includes the use of instrumental deglutition evaluations such as the videofluoroscopy swallowing study (VFSS) and transnasal fibreoptic endoscopic evaluation of swallowing (FEES). Early diagnosis can also make it possible to implement preventive measures, and thus to reduce the risk of complications such as aspiration pneumonia.

To identify the risk of dysphagia in MS, Bergamaschi et al. (2009) developed a simple questionnaire, the DYMUS (Questionnaire for the assessment of Dysphagia in Multiple Sclerosis) (Bergamaschi et al. 2008), validated in 2010 in a large number of patients (Bergamaschi et al. 2009). This instrument is the first questionnaire developed specifically for patients with MS. The DYMUS questionnaire proved to be a useful and consistent instrument to detect oropharyngeal dysphagia and its main features in MS. It can also be used to identify individuals in need of more objective evaluations of swallowing and directing programs for prevention of aspiration. It also showed a very good correlation with swallowing problems and Expanded Disability Status Scales (EDSS) scores. Furthermore, it was associated with a very good internal consistency for both “dysphagia for solid” and “dysphagia for liquid” subscales (Bergamaschi et al. 2008,2009).

The purpose of this study was to translate, cross-culturally adapt and validate to Portuguese the DYMUS questionnaire for the assessment of dysphagia in Multiple Sclerosis.

## Results

In total, 100 patients were included into the validation study. Sixty-nine percent of the patients was female. The mean age of the patients was 42.5 years, with a range between 13 and 69 years. The majority of patients (66.0%) had a relapsing-remitting form of MS. The median disease duration of the participants was 8 years (range: 0–40 years), and their median EDSS score was 3 (range: 0–9). At the time of the interview 15 patients (15.0%) claimed to have swallowing problems (Table 1).

**Table 1:** **Socio**-**demographic and clinical characteristics of the MS patients** (**n**= **100**)

Variables	Values
Gender, N (%)	
Female	69 (69,0)
Male	31 (31,0)
Age (years), N (%)	
< 50 anos	71 (71,0)
50 anos ou mais	29 (29,0)
MS^a^ subtype, N (%)	
Relapsing remitting	66 (66,0)
Primary progressive	18 (18,0)
Secondary progressive	16 (16,0)
Disease duration (years)	
Median	8
Range	(0–40)
EDSS^b^	
Median	3
Range	(0–9)
FS^c^ pyramidal	
Median	2
Range	(0–5)
FS brainstem	
Median	0
Range	(0–4)
FS cerebellar	
Median	0
Range	(0–4)
FS mental	
Median	0
Range	(0–3)
FS sensory	
Median	0
Range	(0–4)
FS visual	
Median	0
Range	(0–3)
FS sphincter	
Median	0
Range	(0–5)

^a^*MS* multiple sclerosis.

^b^*EDSS* expanded disability status scale.

^c^*FS* functional system.

Table 2 shows the percentages of positive responses to the DYMUS single questions. The mean DYMUS score was 1.58 (SD 1.95, range 0–8). The difficulty swallowing solid food (question # 1) and food sticking (question # 4) items showed the lowest frequency of positive responses (≤10%). According to DYMUS-BR, 58,0% of the patients were classified as having dysphagia, 31,0% had dysphagia for liquid food (positive scores on questions 2, 6 or 9) and 53.0% dysphagia for solid food (positive scores on questions 1, 3, 4, 5, 7, 8 or 10); 26.0% had dysphagia for solids and liquids simultaneously. For the 85 patients who reported no difficulty in swallowing by the initial question, 43 (50.6%) had positive responses to the DYMUS questionnaire and 11 of these (12.9%) were classified as having alarming dysphagia. The DYMUS questionnaire detected 3.5 times more dysphagia (53.0% versus 15.0%) than patients’ self-assessment. Regarding the severity of dysphagia, 36.0% of patients were classified as mild dysphagia and 22.0% as alarming dysphagia, while most cases of mild dysphagia (33/36 = 91.7%) had not responded to the initial question “Do you have difficulty swallowing?”. Almost half of alarming cases (10/22 = 45.5%) answered no to that same question. The Cronbach’s alpha value of the DYMUS-BR was estimated to be 0.72, representing a high internal consistency, demonstrating consistency of the questionnaire items after translation to the Portuguese language. Cronbach’s alpha coefficients of the 2 subscales dysphagia for solids and liquid were also calculated: The Cronbach’s alpha of the 7-item dysphagia for solids questionnaire was 0.65 while that of the 3-item dysphagia for liquids questionnaire was 0.67. By analyzing the matrix of inter-item correlations (Table 3), a positive correlation between all items, except question 10 (“weight loss” item), was found. Question 10 had also the lowest correlation coefficient. For the remaining questions (1 a 9) the inter-item correlation coefficients between scores ranged from 0.044 (between “food sticking” and “cough or choking sensation” questions) to 0. 595 (between “difficulty swallowing liquids” and “cough or choking sensation” questions).

**Table 2:** **Percentages of positive responses to the DYMUS**-**BR single questions**

DYMUS-BR questions	N (%)
1. Do you have difficulty swallowing solid food (such as meat, bread and the kike)?	10 (10,0)
2. Do you have difficulty swallowing liquids (such as water, milk and the like)?	17 (17,0)
3. Do you have globus sensation (the feeling of a lump) in your throat when swallowing?	17 (17,0)
4. Does food stick in your throat?	7 (7,0)
5. Do you cough or have a choking sensation after ingesting solid food?	15 (15,0)
6. Do you cough or have a choking sensation after ingesting liquids?	22 (22,0)
7. Do you need to swallow several times before solid foods “goes down” completely?	14 (14,0)
8. Do you need to cut food into small pieces be able to swallow it?	24 (24,0)
9. Do you need to take many sips in order to drink?	14 (14,0)
10. Have you lost weight?	18 (18,0)

**Table 3:** **Reliability analysis based on the DYMUS_BR inter-item correlation**

	Question 1	Question 2	Question 3	Question 4	Question 5	Question 6	Question 7	Question 8	Question 9	Question 10
Question 1	1,000									
Question 2	,204	1,000								
Question 3	,293	,150	1,000							
Question 4	,300	,189	,189	1,000						
Question 5	,420	,332	,257	,434	1,000					
Question 6	,306	,595	,145	,044	,047	1,000				
Question 7	,442	,124	,124	,228	,395	,134	1,000			
Question 8	,281	,369	,182	,213	,420	,323	,313	1,000		
Question 9	,250	,354	,124	,115	,153	,273	,336	,246	1,000	
Question 10	,017	**-,004**	,134	**-,027**	,095	,003	,036	**-,020**	,036	1,000

* In bold, negative correlations.

Construct validity was assessed by using a single-item assessment of perceived dysphagia. Individuals who reported dysphagia in the initial query “*Do you have difficulty swallowing*?” obtained a significantly higher DYMUS-BR score (median 5, range 1 – 8) compared with those not perceiving dysphagia (median 1, range 1–6), supporting the ability of the questionnaire to discriminate between individuals with and without dysphagia (p<0.001). In addition, Spearman’s correlation coefficients were positive (r_s_ = 0,357) and show that there was a significant correlation between the both measures (P value= 0.01), suggesting that the two scales were virtually interchangeable in assessing dysphagia.

## Discussion

Until the year 2008, it had not been described in the scientific literature any assessment tool to access the risk of dysphagia specifically in MS patients. Bergamashi and his coworkers (Bergamaschi et al. 2008,2009) developed and validated in a large population (1,734 patients) the DYMUS questionnaire, the subject of this study.

The translation, cross cultural adaptation and validation of questionnaires must be carefully performed. Beaton et al. (1976) emphasized that a poor translation process can result in a non-equivalent to the original instrument. The lack of equivalence can limit the comparison of results obtained in populations separated by their own languages and cultures. In this study, the careful execution of each of these steps aimed the maintenance of the unique properties of the questionnaire, which was reinforced by conducting a pilot study with 40 MS patients and the participation of the experts committee, ensuring content and comprehensibility validity (or face validity) of the Brazilian version of the questionnaire.

In the validation set, more than half (50.6%) of the 85 patients who reported no difficulty in swallowing in the initial question had positive responses to the DYMUS questionnaire and 12.9% of these were classified as having alarming symptoms of dysphagia, pointing to a significant percentage of silent cases, who were identified only by DYMUS. Although we have no knowledge of another published study using the DYMUS questionnaire by way of discussion, we cite the study of Terré-Bolliart et al. (2004) in which, correlating the clinical and VFSS evaluations, found differences in the results obtained by these different forms to assessment of dysphagia, emphasizing the importance of the association of clinical and instrumental methods to evaluate the swallowing process in patients with MS. Also Weisner et al. (2002) studied patients with MS through VFSS, and found swallowing disorders in 75% of patients without clinical signs and symptoms of dysphagia, demonstrating that even patients without symptoms or complaints of dysphagia may have impairments in swallowing. Leder et al. (1998) detected silent aspiration in half of the patients with different neurological degenerative disorders, including multiple sclerosis. These results point to the importance of early identification of MS patients with risk of dysphagia, especially those without clinically evident symptoms of swallowing disorders.

By applying DYMUS-BR, 58% of patients were classified as having some type of swallowing disorder. In the original study developed by Bergamaschi et al. (2008), the prevalence of dysphagia measured by DYMUS was 35%. On the other hand, in the DYMUS validation process (Bergamaschi et al. 2009), 31% of patients had at least one abnormal response on the questionnaire. Using other methods to determine the prevalence of dysphagia in MS patients, Poorjavad et al. (2010) found that 31.7% of the 101 analyzed patients had dysphagia.

The higher values obtained in our study may be attributed to the absence of obvious clinical signs and symptoms of swallowing disorders.

The final version of the DYMUS-BR questionnaire, whose internal consistency was classified as high (Cronbach’s alpha coefficient 0.730), can be considered as reliable, due to the degree of homogeneity of items. The subscales dysphagia for liquids (Cronbach’s alpha coefficient = 0.673) and dysphagia for solids (Cronbach’s alpha coefficient = 0.670) showed satisfactory internal consistencies. In the original validation of the DYMUS, Bergamaschi et al. obtained, the following coefficients: 0.914 (10-item DYMUS questionnaire), 0.885 (dysphagia for solids) and 0.864 (dysphagia for liquids) (Bergamaschi et al. 2009).

In the original version of the DYMUS questionnaire, the question 10 (do you have weight loss?) Was less correlated to other questions, getting still the second lowest frequency of positive responses (8.3%), but even so it has been kept in the questionnaire.in our study, question 10 was also the only one which was not a positive correlation and low correlation coefficient. this can be explained by the fact that in our study, the mean disease duration of MS patients was 8 years old, with a median EDSS of 3, which shows no or mild degree of disability. The association between weight loss and dysphagia is found in the evolutionary course of neurological disorders, being found more often in advanced stages of the disease, where there is greater functional dependency and difficulty swallowing, which may explain the lower scores on question 10.

To our knowledge, there is only one validation study of the DYMUS questionnaire (Bergamaschi et al. 2009).Because of this, by way of comparison, the results obtained were analyzed in the light of other validation studies for the diagnosis of dysphagia. For the construct validity, Dwivedi et al. (2010) have analyzed a cohort of 31 head and neck cancer patients using the correlation coefficient and Mann–Whitney U-test in the validation process of the Sydney Swallow Questionnaire. Both were statistically significant (P <0.01), as in the present study. In the validation process of the Mayo Dysphagia Questionnaire, Grudell et al. (2007) found excellent Spearman rank correlation coefficients values (range of 0.87-0.98). In another study, Chen et al. (2001) developed and validated a dysphagia-specific quality-of-life questionnaire for head and neck cancer patients with Spearman rank correlation coefficients between 0.21 and 0.52. In the current study the coefficient was 0.357, indicating significant positive correlations (P value = 0.01).

The main limitation of our study was that DYMUS is a screening test and like other screening procedures was designed to identify high-risk patients with a specific problem and has been designed to detect signs of dysphagia and its main characteristics in MS patients. Future studies should be focused to analyze correlations between the DYMUS-BR scores and instrumental tools for evaluations of dysphagia such as Videofluoroscopy or FEES. Studies with larger populations are also needed in order to better analyze the types and characteristics of oropharyngeal dysphagia in Brazilian patients with Multiple Sclerosis.

## Conclusion

In conclusion, the Brazilian version of the DYMUS questionnaire kept the features originally described, demonstrating to be a reliable, valid, consistent and easy tool to detect the risk of dysphagia in MS patients, that can also be used by healthcare professionals to pre-select patients who could be submitted for more specific instrumental analyses, and to address to programs for prevention of aspiration.

## Method

### Study sample

A sample of MS patients based on the McDonald criteria (McDonalds et al. 2001) in the clinical types Relapsing-Remitting (RRMS), Primary Progressive (PPMS) and Secondary Progressive (SPMS), who were visited at the Multiple Sclerosis outpatient Clinic of Hospital of Lagoa, Rio de Janeiro, Brazil, were recruited to this study between August 2009 and June 2010.

Patients with other idiopathic demyelinating diseases; patients with no confirmed MS diagnosis; patients in an acute relapse; and patients incapable of verbal communication were excluded from this study. The authors of the original version of the DYMUS questionnaire authorized the translation and cross-cultural adaptation to the Portuguese language. The study was approved by the Ethics and Research Committee of Federal University of the State of Rio de Janeiro –UNIRIO (n^o^ 0036.000-09) and all patients signed the informed consent form.

### Instrument

The DYMUS is a self-reported questionnaire composed by 10 questions that evaluate two dimensions: dysphagia for solids (positive scores in questions 1, 3, 4, 5, 7, 8 or 10) and dysphagia for liquids (positive scores in questions 2, 6 or 9). All the answers are dichotomous, coded as 1 or 0, depending on the presence or the absence of the event. The total score is achieved by the sum of the two dimensions, ranging from 0 to 10 points. Dysphagia is identified by at least one abnormal response and is considered alarming when the score is equal to or higher than three (Bergamaschi et al. 2008,2009).

At the time of the examination all the patients were asked whether they had swallowing problems and after that, irrespective of their response, the DYMUS questionnaire was administered by a trained speech pathologist.

The Expanded Disability Status Scale (FS/EDSS) (Kurtzke 1983) was used as a standardized neurological examination. The EDSS assesses neurological impairment and disability and was performed by a trained doctor. EDSS score varies between 0 and 10, a high score indicates a more severe disability (Sonder et al. 2012; Kurtzke 1983).

### Translation, back-translation and cross-cultural adaptation of questionnaire

The translation, including forward and backward translations, and the cross-cultural adaptation of the DYMUS questionnaire were carried out according to international standards. The criteria proposed by Guillemin et al. (1993) and Beaton et al. (1976) we used, fulfilling the 5 steps for translation and cultural adaptation. The original version of the DYMUS questionnaire was translated to Portuguese by two Brazilian translators who were fluent in English, one being a health professional (speech and language therapist) and the other with no technical/scientific knowledge of the topic under study. The two translations were compared by the researcher in charge and the required adjustments were subsequently made with the consent of both translators resulting into a single version of the translation. This version was back translated into English by two bilingual translators, whose mother tongue is English, who had no access to the original English version of the questionnaire, thus preserving its ambiguity. The backward translation was compared and considered equivalent to the original version.

In the second phase of the study, comprehensibility and content validity of the questionnaire were tested using cognitive interviews with 40 MS patients (pilot study). In order to improve the intelligibility of the instruments when necessary and optimize the face and content validity for the main study, each patient was asked to explain what had understood about each question.

A committee composed by four health professionals with experience in neurology and dysphagia (one speech therapist, two neurologists and one physical education), bilingual, revised the produced versions in light of the difficulties presented by patients, making the semantic and idiomatic modifications as necessary. Questions 1, 2, 5, 8 and 10 were comprised of 100% of patients. Based on the information obtained, adjustments had to be made to questions 3, 4, 6, 7 and 9, besides substituting the words “deglute and deglutition” for “swallow”, given that those words were not understood by the patients. Furthermore, one patient had difficulty understanding the question 3 (2.5%), two (5.0%) in question 4 and 6, three (7.5%) on question 9 and 11 in question 7 (27.5%).

The definitive Brazilian version, named DYMUS-BR, was produced after the face and content validity results in the pilot study had been approved by this committee. The final version of the questionnaire was maintained with 10 questions, similar to the original version (Additional file 1).

### Reliability and construct validity

To evaluate the reliability of the DYMUS-BR questionnaire we computed Cronbach alpha (Cronbach 1951), which is a reliability coefficient based on internal consistency. The internal consistency was estimated by Cronbach’s alpha coefficient for dysphagia as a whole and for dysphagia for solid and liquid. The Cronbach’s alpha coefficient was analyzed according to the Instrument Review Criteria (SAC): between 0 to 0.6 unsatisfactory reliability, between 0.6 to 0.7 satisfactory reliability, and between 0.7 to 1.0 high reliability (Scientific Advisory Committee of the Medical Outcomes Trust – SAC Instrument Review Criteria 1995). A questionnaire can be considered reliable if obtains values between 0.65 and 1 (Scientific Advisory Committee of the Medical Outcomes Trust – SAC Instrument Review Criteria 1995). In order to evaluate item homogeneity we computed the inter-item correlation matrix. Construct validity was evaluated by testing the operationalization of the measure dysphagia of the questionnaire against the patients’ self-assessment of dysphagia, used as a proxy, measured by the response to the initial question “Do you have difficulty swallowing?”. To achieve this, a cross-sectional study was performed. As recommended by (Dawson & Trapp 2004) the minimum number of patients to be tested should correspond to 10 patients for each item of the questionnaire to be validated. As DYMUS questionnaire has ten items, the inclusion of 100 patients was sufficient. Mann–Whitney U test was used to determine if differences existed and Spearman’s rank correlation coefficient (r_s_) was used to assess the significance of the association between DYMUS and patient’s self-assessment.

### Data analysis

The descriptive analysis was conducted for characterization of the socio-demographic and clinical data of the patients. Categorical variables were shown as percentages. Continuous variables were shown as average or mean along with the respective standard deviation or percentile. The answers to the DYMUS-BR questionnaire were grouped into: without dysphagia (zero score) and dysphagia (1 or more abnormal response). Patients with dysphagia were classified as: dysphagia for solids (positive scores on questions 1, 3, 4, 5, 7, 8 or 10) or: dysphagia for liquids (positive scores on questions 2, 6 or 9). Moreover, dysphagia was classified as mild (1 or 2 abnormal responses) or alarming (3 or more abnormal responses) (Bergamaschi et al. 2008,2009). Cronbach’s alpha coefficient, inter-item correlation matrix, Mann–Whitney U test and Spearman’s rank correlation coefficient were used. A significance level of 0.05 was set. Data analyses were performed using *Statistical Package for Social Sciences* (*SPSS*), version 14.0, 2006 for windows software.

## Authors’ information

Déborah S Sales - Speech and language Pathologyst, master in neurology, Doctor degree in Neuroloy - Federal University of the State of Rio de Janeiro (UNIRIO), Rio de Janeiro, RJ, Brazil.

Regina MP Alvarenga – MD, responsible for the Pós Graduate Program in Neurology - Federal University of the State of Rio de Janeiro (UNIRIO), Rio de Janeiro, RJ, Brazil.

Claudia CF Vasconcelos - MD, Pós Doctor degree Neurology. - Federal University of the State of Rio de Janeiro (UNIRIO), Rio de Janeiro, RJ, Brazil.

Roberta G Silva - Department of Speech and language Pathology, Paulista Estadual University - Marília Campus, São Paulo, Brazil.

Luiz CS Thuler – MD, Coordination of Clinical Research -National Cancer Institute – INCA; Federal University of the State of Rio de Janeiro (UNIRIO) Rio de Janeiro, RJ, Brazil.

## Electronic supplementary material

Additional file 1: **The English original version of the DYMUS questionnaire and the Brazilian final version of the DYMUS-BR questionnaire.** (DOC 34 KB)
