# Supplementary material for: Translation, cross-cultural adaptation and validation of the Portuguese version of the DYMUS questionnaire for the assessment of dysphagia in multiple sclerosis
Source: Springerplus. 2013 Jul 22;2:332. doi: 10.1186/2193-1801-2-332 (PMC3728525; doi:10.1186/2193-1801-2-332)
Supplement: Supplementary file 1 — Additional file 1: The English original version of the DYMUS questionnaire and the Brazilian final version of the DYMUS-BR questionnaire. (DOC 34 KB) [file 40064_2013_405_MOESM1_ESM.doc]

Additional file 1 - The English original version of the DYMUS questionnaire and the Brazilian final version of the DYMUS-BR questionnaire

| DYMUS  original version | DYMUS-BR  Brazilian final version |
| --- | --- |
| 1. Do you have difficulty swallowing solid food (such as meat, bread and the like)? | 1. Você tem dificuldade para engolir alimentos sólidos (tais como carne, pão e similares)? |
| 2. Do you have difficulty swallowing liquids (such as water, milk and the like)? | 2. Você tem dificuldade para engolir líquidos (tais como água, leite e similares)? |
| 3. Do you have a globus sensation (the feeling of a lump) in your throat when swallowing? | 3. Você tem a sensação de bolo na garganta enquanto engole? |
| 4. Does food stick in your throat? | 4. Você tem comida grudando na sua garganta? |
| 5. Do you cough or have a choking sensation after ingesting solid food? | 5. Você tosse ou tem uma sensação de asfixia após ingerir sólido? |
| 6. Do you cough or have a choking sensation after ingesting liquids? | 6. Você tosse ou tem uma sensação de asfixia após ingerir líquido? |
| 7. Do you need to swallow several times before solid food “goes down” completely? | 7. Você precisa engolir varias vezes para engolir completamente os alimentos sólidos? |
| 8. Do you need to cut food into small pieces be able to swallow it? | 8. Você precisa cortar a comida em pequenos pedaços antes de engolir? |
| 9. Do you need to take many sips in order to drink? | 9. Você precisa tomar vários goles para engolir completamente os líquidos? |
| 10. Have you lost weight? | 10. Você tem perdido peso? |
